# Supplementary figures and images for: Reanalysis of cryo-EM data reveals ALK-cytokine assemblies with both 2:1 and 2:2 stoichiometries
Source: PLoS Biol. 2025 Apr 10;23(4):e3003124. doi: 10.1371/journal.pbio.3003124 (PMC12017499; doi:10.1371/journal.pbio.3003124)

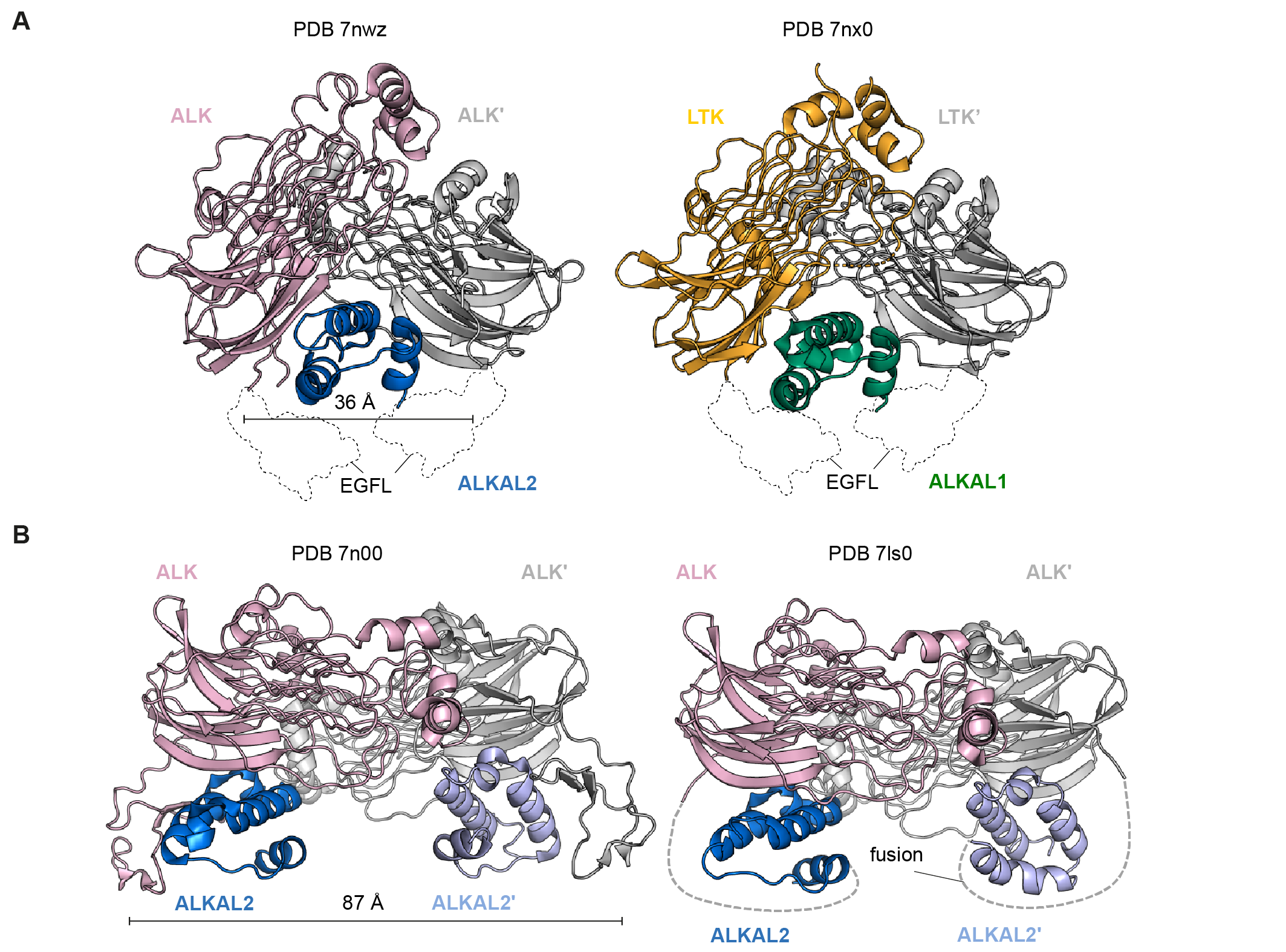

Supplement: S1 Fig — (A) Cartoon views of structures of ALK-ALKAL2 and LTK-ALKAL1 complexes with 2:1 stoichiometry as determined by De Munck and colleagues [5] (PDB entries 7nwz and 7nx0) and (B) of ALK-ALKAL2 with 2:2 stoichiometry as determined by Reshetnyak and colleagues [7] and Li and colleagues [6] (PDB entries 7n00 and 7ls0). Putative positions of the EGFL domains absent in (A) are shown as dotted outlines. (TIF) [file pbio.3003124.s001.tif]

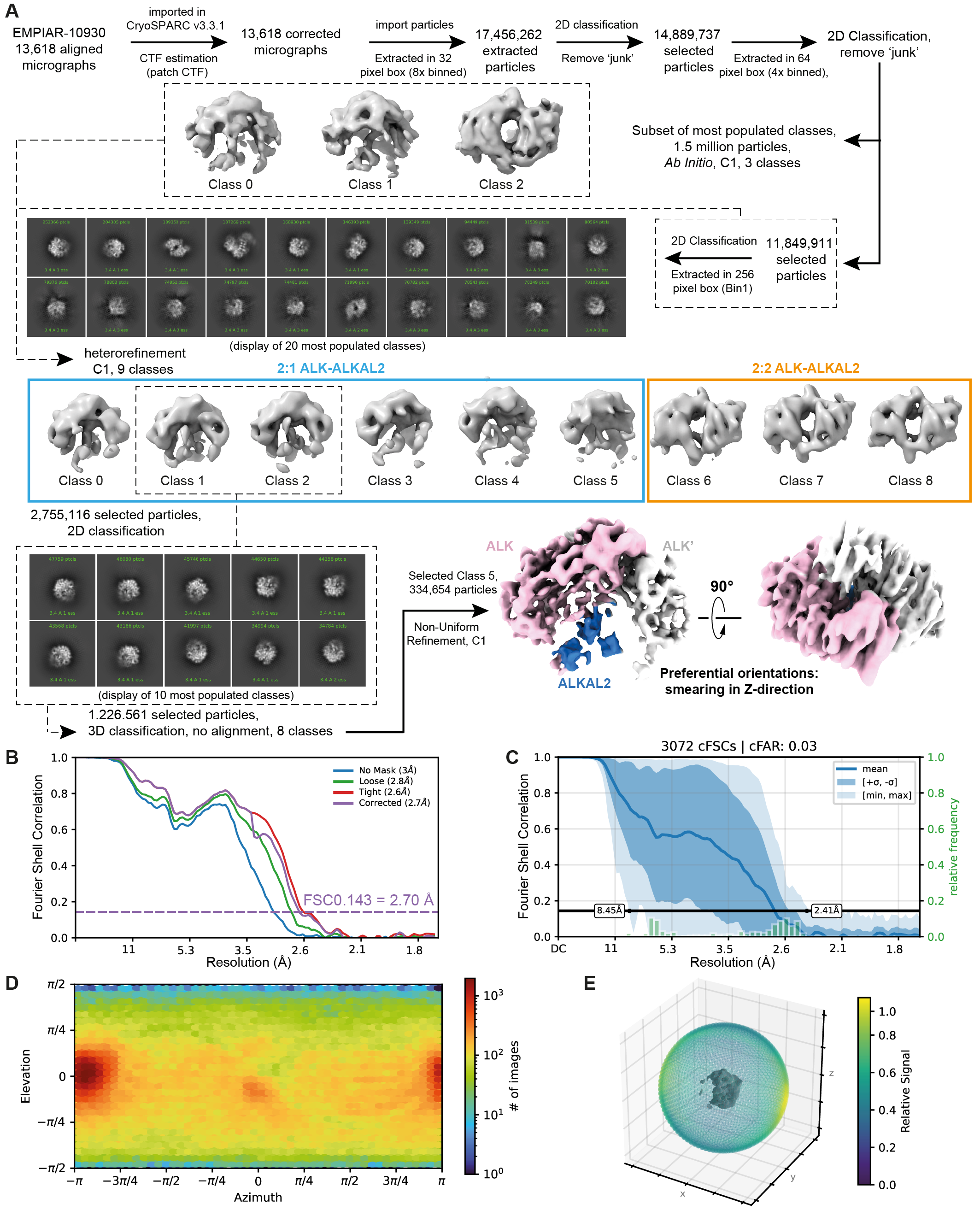

Supplement: S2 Fig — (A) All initial processing steps were performed in CryoSPARC [18] v3.3.1. (B) Fourier Shell Correlation (FSC) plot corresponding to the final map obtained after non-uniform refinement (C1 symmetry). Curves are shown after applying no mask (blue), a loose mask (green), or a tight mask (red) to the two half-maps before calculating FSCs. The corrected FSC (purple) is calculated using the tight mask with correction by noise substitution [20], and the resolution at FSC = 0.143 is annotated via a dotted purple line. (C) Conical FSC summary plot generated via “Orientation Diagnostics” in cryoSPARC v4.5. (D) Left: Azimuth plot showing the distribution of orientations over Azimuth (x-axis) and Elevation (y-axis) angles for the particle set corresponding to the NU-refined map shown in (A). Right: Plot showing Relative Signal amount vs. Viewing Direction. (TIF) [file pbio.3003124.s002.tif]

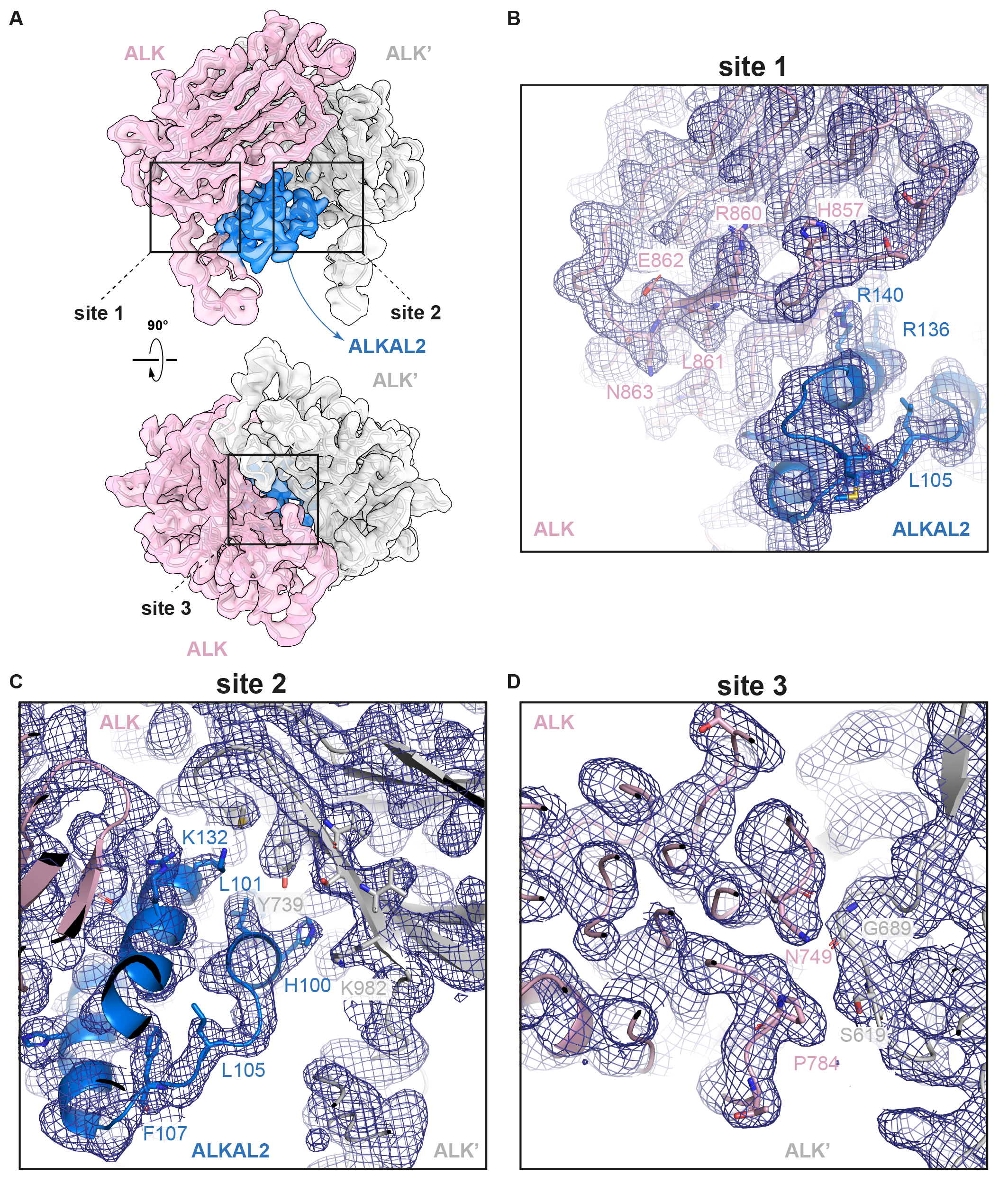

Supplement: S3 Fig — (A) Front (upper view) and top (lower view) views are shown of a transparent map of the 2:1 ALK-ALKAL2 complex with fitted structural model, with ALK colored pink/gray and ALKAL2 colored blue. Insets shown in (B), (C), and (D) are annotated with black squares. (B and C) Insets showing zooms of the site 1 ALK-ALKAL2 (B), site 2 ALK’-ALKAL2 (C), and site 3 ALK-ALK’ (D) interfaces, with the EM map displayed as a dark blue mesh. The fitted 2:1 ALK-ALKAL2 model is shown as a cartoon with selected annotated residues shown as sticks (ALK colored pink/gray and ALKAL2 colored blue). (TIF) [file pbio.3003124.s003.tif]

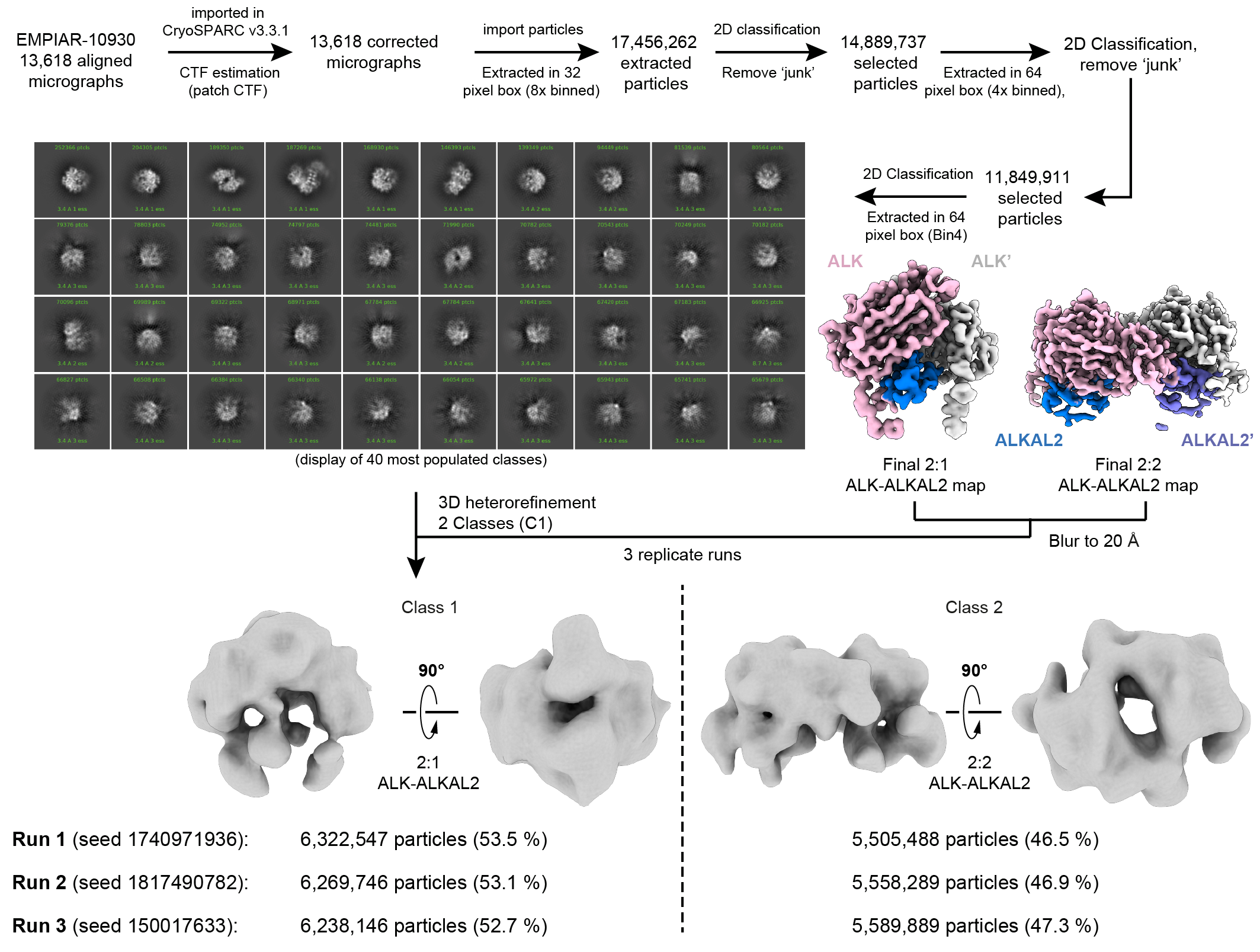

Supplement: S4 Fig — All initial processing steps were performed in CryoSPARC [18] v3.3.1. The three 3D heterorefinement replicate runs, each ran with a different random seed, were performed using CryoSPARC v4.5. (TIF) [file pbio.3003124.s004.tif]

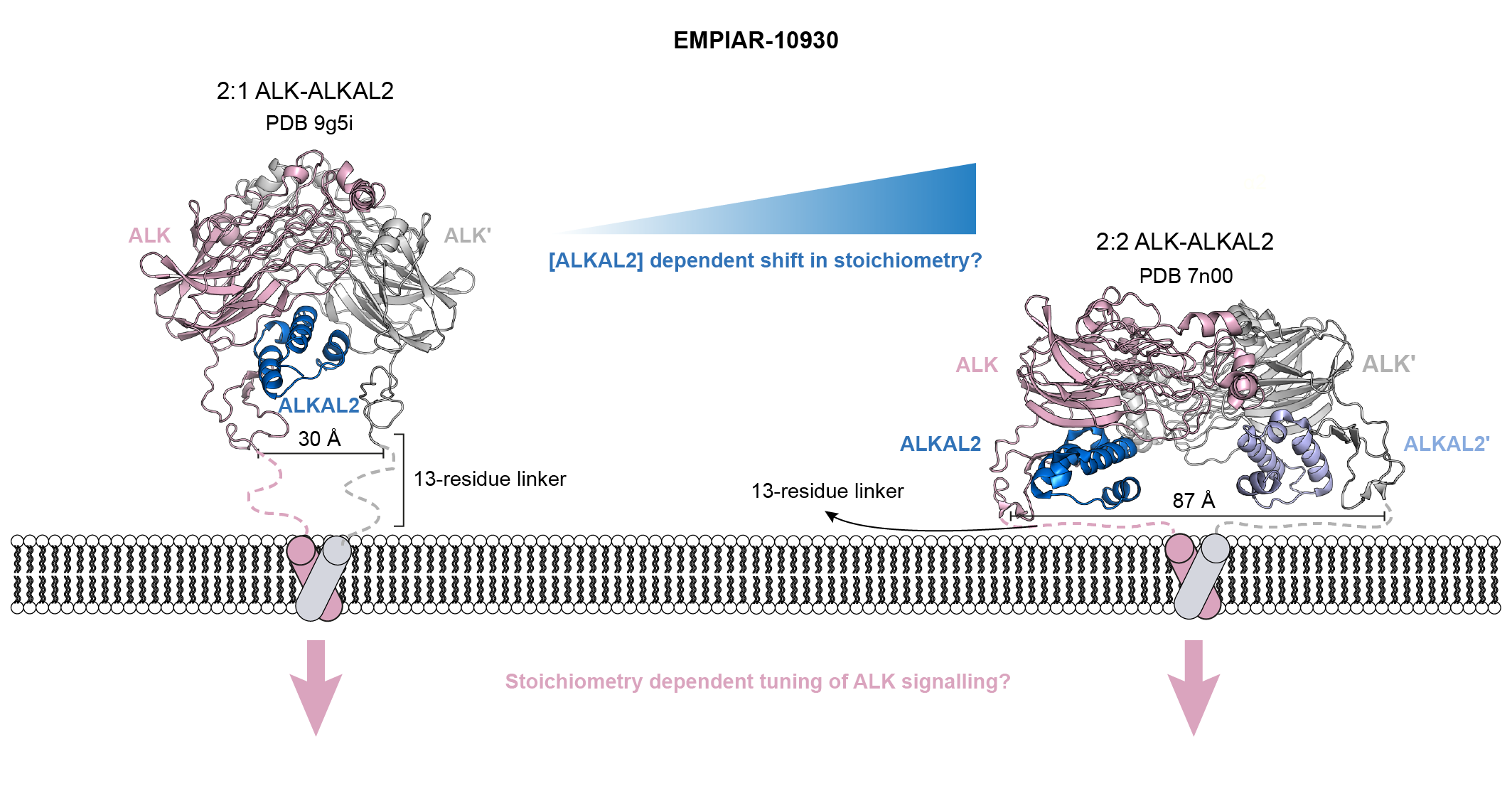

Supplement: S5 Fig — Models with pdb code 9g5i (this study) and 7n00 (Reshetnyak et al., 2021) [7] are shown as cartoons with ALK/ALK’ colored pink/gray and ALKAL2 colored blue in the 2:1 ALK-ALKAL2 complex, or blue/purple in the 2:2 ALK-ALKAL2 complex. The distance between the C-termini of membrane-proximal EGFL domains is annotated. (TIF) [file pbio.3003124.s005.tif]

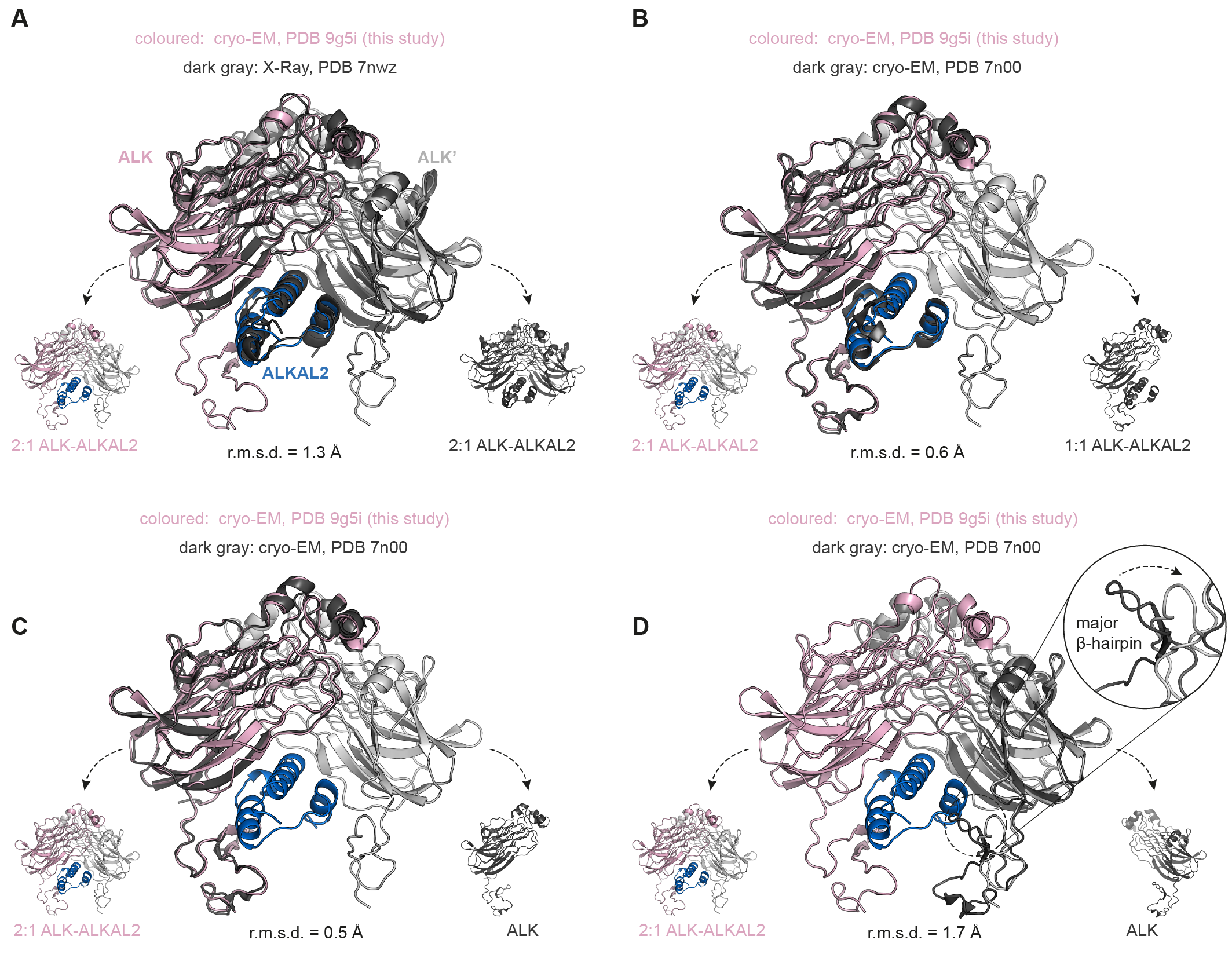

Supplement: S6 Fig — (A) Structural alignment of the 2:1 ALK-ALKAL2 X-ray structure (PDB 7nwz) with the cryo-EM structure of the 2:1 ALK-ALKAL2 complex obtained in this study (PDB 9G5I). (B) Structural alignment of a 1:1 ALK-ALKAL2 complex, extracted from the cryo-EM structure of the 2:2 ALK-ALKAL2 complex (PDB 7n00), with the cryo-EM structure of the 2:1 ALK-ALKAL2 complex obtained in this study. (C) Structural alignment of one copy of ALK, extracted from the cryo-EM structure of the 2:2 ALK-ALKAL2 complex (PDB 7n00), with the first ALK molecule (pink) present in the cryo-EM structure of the 2:1 ALK-ALKAL2 complex obtained in this study. (D) Structural alignment of one copy of ALK, extracted from the cryo-EM structure of the 2:2 ALK-ALKAL2 complex (PDB 7n00), with the second ALK molecule (ALK’, gray) present in the cryo-EM structure of the 2:1 ALK-ALKAL2 complex obtained in this study. (TIF) [file pbio.3003124.s006.tif]
