# Supplementary material for: Reanalysis of cryo-EM data reveals ALK-cytokine assemblies with both 2:1 and 2:2 stoichiometries
Source: PLoS Biol. 2025 Apr 10;23(4):e3003124. doi: 10.1371/journal.pbio.3003124 (PMC12017499; doi:10.1371/journal.pbio.3003124)
Supplement: S1 Table — (DOCX) [file pbio.3003124.s007.docx]

**Supplementary Table 1.** Cryo-EM data collection, processing, refinement and validation statistics

|  | 2:1 ALK_TG-EGFL_-ALKAL2 |
| --- | --- |
| Data Collection (EMPIAR-10930, Reshetnyak *et al*. 2021[7]) |  |
| Magnification | 15,000 |
| Voltage (kV) | 300 |
| Electron exposure (e^–^/Å^2^) | 88.4 |
| Defocus range (μm) | 0.8 – 1.8 |
| Pixel size (Å)  Micrographs | 0.826  13,618 |
| Data Processing (This study)  Symmetry imposed | C1 |
| Initial particle images (no.) | 18,053,705 |
| Final particle images (no.) | 142,986 |
| Map resolution (Å)  FSC threshold | 3.2  0.143 |
| Map resolution range (Å) | 3.0 – 5.5 |
|  |  |
| Refinement (This study) |  |
| Initial model used (PDB code) | 7N00 |
| Model resolution (Å)  FSC threshold | 3.2  0.5 |
| Map sharpening B factor (Å^2^) | -100 |
| Model composition  Non-hydrogen atoms  Protein residues | 5,252  732 |
| B factors (Å^2^)  Protein | 165.45 |
| R.m.s. deviations  Bond lengths (Å)  Bond angles (°) | 0.009  1.193 |
| Validation  MolProbity score  Clashscore  Poor rotamers (%)  CaBLAM outliers (%)  CC (mask/volume) sharpened, unsharpened  EMRinger  (sharpened/unsharpened) | 1.86  9.85  0.00  2.64  0.76/0.71, 0.80/0.88  2.10/1.51 |
| Ramachandran plot  Favored (%)  Allowed (%)  Disallowed (%)  Ramachandran Z-score  Whole  Helix  Sheet  Loop | 95.04  4.96  0.00  -1.92  -1.17  -1.29  -1.25 |
