# Supplementary material for: Reanalysis of cryo-EM data reveals ALK-cytokine assemblies with both 2:1 and 2:2 stoichiometries
Source: PLoS Biol. 2025 Apr 10;23(4):e3003124. doi: 10.1371/journal.pbio.3003124 (PMC12017499; doi:10.1371/journal.pbio.3003124)
Supplement: Attachment — Submitted filename: PLOS_Biology_Short_Report_Felix_et_al_2025_Rebuttal_FINAL.docx [file pbio.3003124.s011.docx]

**Short Report – Response to Reviewers’ comments**

**Reanalysis of cryo-EM data reveals ALK-cytokine assemblies with both 2:1 and 2:2 stoichiometries**

Jan Felix^1,2*^, Steven De Munck^1,2^, J. Fernando Bazan^3^, Savvas N. Savvides^1,2*^

^1^VIB-UGent Center for Inflammation Research, 9052 Ghent, Belgium.

^2^ Unit for Structural Biology, Department of Biochemistry and Microbiology, Ghent University, 9052 Ghent, Belgium.

^3^ *ħ* bioconsulting llc, Stillwater, MN, USA.

*Correspondence: [savvas.savvides@ugent.be](mailto:savvas.savvides@ugent.be) ; jan.felix@ugent.be

**Response to Reviewers’ Comments**

We thank all four reviewers for their expert assessment of our work and constructive input, and their strong endorsement of our study for publication in PLOS Biology. Here, we provide point-by-point responses (in blue) under each relevant comment. Corresponding changes in the revised version of the manuscript are highlighted in yellow.

**Reviewer #1:**

Felix et al. reprocessed the cryo-EM dataset of the ALK-ALKAL2 complex, which was deposited at EMPIAR. By employing blush regularization, a new feature in RELION5, the authors successfully resolved the structure of the 2:1 ALK-ALKAL2 complex at an overall resolution of 3.2 Å. Previously, this structure could not be determined due to the particles' strong preferred orientation. The resolved 2:1 ALK-ALKAL2 complex is virtually identical to the previously determined crystal structure, supporting the notion that this stoichiometry represents a physiologically relevant state. Therefore, this study provides valuable insights into the activation mechanism of the ALK receptor. The quality of the reported cryo-EM structure is high, and the model appears to be reliable. I recommend its publication as a short report in PLOS Biology.

We thank the reviewer for the positive assessment of our work and appreciation of the quality of the cryo-EM (re-)analysis, and endorsing our manuscript for publication in PLOS Biology.

I have one suggestion for the authors to consider: The paper mentions a hypothesis that increasing local cytokine concentrations might shift an ALK-ALKAL2 assembly from a 2:1 to a 2:2 stoichiometry, increasing the distance between the membrane-proximal domains from 30 Å to 90 Å, potentially leading to different signaling outcomes. This intriguing idea can be easily tested. Can the authors examine ALK activation in response to varying concentrations of ALKAL2? If the hypothesis is correct, a bell-shaped dose-response curve might be observed.

To respond to this suggestion, we refer the reviewer to Reshetnyak *et al.*, PNAS, 2015 ([www.pnas.org/cgi/doi/10.1073/pnas.1520099112](http://www.pnas.org/cgi/doi/10.1073/pnas.1520099112)), in which the authors have actually performed a version of the proposed experiment, which indicated that ALK indeed may display a bell-like signal of activation that peaks at around 1.7 nM ALKAL2 ligand and decreases at higher concentrations (AUG-α synonymous with ALKAL2 in Fig. 2D – top panel, and in Fig. S2A). However, this phenomenon could also be caused by the formation of 1:1 ALK-ALKAL2 complexes at the cell surface, which do not necessarily need to assemble into 2:2 ALK-ALKAL2 complexes. Therefore, we feel this suggestion demands a much more rigorous experimental approach. To this end, we have recently initiated a new study that will employ single molecule co-tracking experiments on live cells in conjunction with complementary cellular studies similar to what has been proposed by the reviewer to provide the full context of the relative abundance of the 2:1 and 2:2 for both cytokine-ALK and the related cytokine-LTK assemblies. Since this is a major study that is part of continued research regarding ALK/LTK in our lab, we feel that it would not be opportune to pre-empt these studies in this manuscript and hope the answer above is sufficient as a response.

**Reviewer #2 [identifies himself as Moosa Mohammadi]:**

In this manuscript, Felix and colleagues seek to address the ongoing debate regarding mechanism of ligand-driven dimerization/activation of ALK receptor tyrosine kinase family members, ALK and LTK. Previously, this group reported X-ray crystal structures of ALK and LTK ectodomains complexed with their cognate ligands, ALKAL2, and ALKAL1, respectively, revealing how a single ligand induces asymmetric receptor dimerization with 1:2 ligand-receptor stoichiometry reminiscent of the growth hormone paradigm. In contrast, based on the cryoEM analysis of the ALKAL2-ALK complex, Reshetnyak and colleagues proposed a symmetric 2:2 model of dimerization for ALK activation. Similarly, Li et al., reported the X-ray structure of a 2:2 ALKAL2-ALK symmetric dimer using an ALK-ALKAL2 fusion protein. To understand the discord between these structures, Felix and colleagues reanalyzed the raw cryo-EM data deposited by Reshetnyak et al. (access code EMPIAR-10930) and found that the bulk (~60%) of ALKAL2-ALK particles arrange as 1:2 complexes as seen in 2D classification. However, these particles suffer from severe preferential particle orientations leading to highly anisotropic maps which preclude model building and detailed structural interpretations. The authors mitigated this problem by performing exhaustive automatic particle orientation rebalancing as implemented in the latest version of cryoSPARC to trim over-populated views, followed by 3D refinement with Blush regularization within Relion. As a result, the authors were able to achieve a dramatically improved map at 3.2 Å resolution allowing them to confidently build a 1:2 ALKAL2-ALK model resembling that seen in their previous X-ray structures.

This study has been conducted meticulously and clearly demonstrates that the asymmetric mode of receptor dimerization is a common mechanism by which ALK/LTK signaling is initiated at the cell surface. The 2:2 symmetric dimer seen in EMPIAR-10930 dataset is likely due to the presence of saturating ligand concentration in the sample preparation.

We thank the reviewer for the constructive comments and endorsement of our study. We provide below point-by-point responses to the comments raised by the reviewer.

Comments:

1)	Page 4, line 139: A comparable distance (~30-40 Å) is seen between the membrane insertion points of the two extracellular domains of FGF receptor chains in the recently published cryoEM structures of three distinct 1:2:1:1 FGF23-FGFR-αKlotho-HS quaternary assemblies (Chen et al., Nature 2023). Notably, these structures feature asymmetric mode of receptor dimerization by the ligand. This should be cited.

Indeed, it would be appropriate to also reference the paper describing the FGF23–FGFR–αKlotho–HS quaternary complex, and have now added this reference to the paragraph that lists RTK-cytokine assemblies with membrane proximal ends spaced 30-40 Å apart (**Lines 173 – 179**):

‘Despite this asymmetry, the C-termini of the EGFL domains of the two ALK copies in the 2:1 ALK_TG-EGFL_-ALKAL2 complex obtained from EMPIAR-10930 are spaced ˜30 Å apart (**Figure 1**), a distance that would allow facile juxtapositioning of receptor chains and TM helices as already illustrated for many RTK—cytokine assemblies featuring cytokine-mediated receptor dimerization such as KIT–SCF[10, 11], CSF-1R–CSF-1[12], Flt3–Flt3R[13], VEGF-VEGFR[16], PDGF-PDGFR[15], EGF–EGFR[14], FGF23–FGFR–αKlotho–HS [9], HER2–HER3–NRG1β[20] and Insulin bound to insulin receptor (IR) family receptors[21, 22].’

2)	Page 4, line 144: the typo "tot" should be corrected to "to".

Thank you for noticing this typo, which is now corrected in the main text.

3)	The authors should briefly mention the adoption of symmetric and asymmetric modes of receptor dimerization by different RTK families, i.e., asymmetric (ALK, FGFR) and symmetric (EGFR, Kit, PDGFR, VEGFR).

We have added the following to the second paragraph of the introduction section of the manuscript (**Lines 40 – 48**):

‘In accordance with the evolutionary relatedness[8] and structural conservation between ALK/LTK, key receptor-receptor and receptor-ligand interactions are conserved in the resulting 2:1 stoichiometric ALK-ALKAL2 and LTK-ALKAL1 assemblies[5], which are pseudo-symmetric due to the asymmetric nature of the ALKAL2/1 ligands. Thus, these structures suggest an asymmetric mode of ALK/LTK receptor dimerization and activation featuring distinct receptor interfaces akin to fibroblast growth factor receptor (FGFR) signaling[8], and contrasting with symmetric receptor dimerization as observed for the RTKs Kit[9, 10], colony stimulating factor 1 receptor (CSF-1R)[11], Flt3 receptor (Flt3R)[12], epidermal growth factor receptor (EGFR)[13] , platelet-derived growth factor receptor PDGFR[14] and vascular endothelial growth factor receptor (VEGFR)[15].’

We have also added the word ‘symmetric’ to line 51 (‘… reported symmetric assemblies obeying a 2:2 stoichiometry …’) to make the distinction between asymmetric (pseudo-symmetric) 2:1 and symmetric 2:2 ALK-ALKAL2 complexes.

**Reviewer #3 [identifies himself as Zhe Zhang]:**

Recent structural studies have shown different stoichiometries for ALK/LTK-cytokine complexes. Specifically, the authors' lab previously documented a 2:1 stoichiometry for ALK-ALKAL2 and LTK-ALKAL1 complexes, while two other groups reported a 2:2 stoichiometry for ALK-ALKAL2 complex. This discrepancy prompted the authors to reanalyze the cryo-EM data deposited in EMPIAR-10930, initially reported by Reshetnyak et al. to generate the 2:2 ALK-ALKAL2 complex structure, in order to resolve these differences.

The authors discovered that the dataset contained two distinct populations of particles corresponding to the 2:1 and 2:2 ALK-ALKAL2 complexes. Notably, the 2:1 complex exhibited severe preferred particle orientations, resulting in anisotropic maps. To address this issue, the authors utilized orientation rebalancing in cryoSPARC to trim overpopulated views, followed by 3D refinement using Blush regularization in RELION. These methods significantly improved the isotropy of the map, yielding a final 3.2 Å resolution map for the 2:1 ALK-ALKAL2 complex. This new cryo-EM structure provides clear evidence for the presence of the 2:1 stoichiometry alongside the previously reported 2:2 stoichiometry in the EMPIAR-10930 dataset. The existence of both stoichiometries suggests that ALK signaling may involve dynamic changes in receptor-cytokine interactions, potentially influenced by local cytokine concentrations.

Overall, this study addresses the previously conflicting structural data and highlights the importance of public data deposition for reanalysis. I am pleased to support its publication in PLOS Biology. I have a few suggestions for the authors to consider, which could further enhance the rigor of this work.

We thank the reviewer for the constructive comments and strong endorsement of our manuscript for publication in PLOS Biology.

1. The authors identified the 2:1 and 2:2 ALK-ALKAL2 complexes solely based on the 2D classification results and concluded that these two populations represent 60% and 40% of the total particles, respectively. However, the reliability of this ratio is questionable. Firstly, it is uncertain whether the two populations can be confidently distinguished based on the blurry 2D images. Secondly, the repeatability of this result is also in doubt. The authors could consider performing a heterogeneous refinement in cryoSPARC using all the good 2D classes as input and taking the two high-resolution 3D models (2:1 and 2:2) as references. In this way, all the particles could be assigned to these two classes according to their similarity to these two structures. If the ratio remains consistent after several repeats, it would further support the authors' conclusion and provide a better evaluation of the ratio between the 2:1 and 2:2 complexes in this dataset.

As suggested by the reviewer, we have now performed three independent heterogeneous 3D refinement runs using the full set of 11.8 million remaining particles after removing junk classes during initial 2D classification, while utilizing the final 2:1 and 2:2 ALK-ALKAL2 complex maps blurred to 20 Å as reference input volumes (see new **Supplementary Figure 4**). We also made sure to use a different random seed for each run (run 1 random seed: 1740971936, run 2 random seed: 1817490782, run 3 random seed: 150017633). The three heterogeneous 3D refinement runs resulted in 53.5 %, 53.1 % and 52.7 % particles corresponding to the 2:1 ALK-ALKAL2 3D class, and 46.5 %, 46.9 % and 47.3 to the 2:2 ALK-ALKAL2 3D class, corroborating the results observed during 2D classification. We have added following paragraph to the results section of the main text (**Lines 116 – 124**):

‘To verify that more than half of the ALK-ALKAL2 particles present in the EMPIAR-10930 dataset indeed correspond to particles with a 2:1 stoichiometry, we pursued three independent heterogeneous 3D refinement runs in cryoSPARC using the full set of 11.8 million particles obtained after removing junk classes during the first 2D classification run (**Materials and Methods**), and utilizing the final 2:1 and 2:2 ALK-ALKAL2 complex maps blurred to 20 Å as reference input volumes (**Supplementary Figure 4**). These three runs, each started using different generated random seeds, resulted in 53.5 %, 53.1 % and 52.7 % particles corresponding to the 2:1 ALK-ALKAL2 3D class, and 46.5 %, 46.9 % and 47.3 % to the 2:2 ALK-ALKAL2 3D class, thus corroborating the results observed during initial 2D classification.’

Furthermore, we added following lines to the Materials and Methods section (**Lines 254 – 258**):

‘To obtain the ratio of particles in the dataset corresponding to 2:1 or 2:2 ALK-ALKAL2 complexes, a 3D heterorefinement run (CryoSPARC v4.5) was performed in triplicate, using the final maps for the 2:1 and 2:2 ALK-ALKAL2 complexes blurred to 20 Å and cleaned particle stack of 11,849,911 particles as input. Each run was initialized using a different random seed (run 1: 1740971936, run 2: 1817490782, run 3: 150017633).’

2. As noted in the Discussion section, the RMSD between the new 2:1 ALK-ALKAL2 complex described in this study and the previously reported 2:1 crystal structure (7nwz) is 1.3 Å. Please include a superimposition of these two structures in the supplementary figure to substantiate this claim. If there are significant differences between these structures, it would be advisable to highlight and briefly discuss them.

We have added a new **Supplementary Figure 6** to the manuscript, depicting structural alignments of ALK-ALKAL2 X-ray and cryo-EM models, along with an extra paragraph describing the differences between the 2:1 and 2:2 cryo-EM models obtained from the EMPIAR-10930 dataset (**Lines 161 – 172)**:

‘The membrane proximal EGFL domain of ALK engaged in the ‘site 1’ interaction adopts a conformation identical to ALK found in the 2:2 ALK_TG-EGFL_-ALKAL2 complex (r.m.s.d. = 0.554 Å over 345 aligned Cα-atoms, **Supplementary Figure 6c**). Likewise, a 1:1 ALK_TG-EGFL_-ALKAL2 binary complex extracted from the 2:2 ALK_TG-EGFL_-ALKAL2 structure matches the ‘site 1’ interface found in the 2:1 ALK_TG-EGFL_-ALKAL2 cryo-EM model (r.m.s.d. = 0.631 over 400 aligned Cα-atoms, **Supplementary Figure 6b**). Due to the asymmetric nature of the ALKAL2 monomer, the EGFL domain of the opposing ALK protomer (ALK’) in the 2:1 ALK_TG-EGFL_-ALKAL2 complex does not make any direct contacts at the ‘site 2’ interaction with ALKAL2 (**Figure 1**). As a result, the ALK’ EGFL domain is less resolved at its extremity due to increased flexibility, resulting in the absence of density for the minor β-hairpin in the cryo-EM map (**Figure 1, Supplementary Figure 6**). Moreover, the major β-hairpin of ALK’ has an outwards tilted conformation compared to the EGFL domain of ALK engaged in the ‘site 1’ interaction (r.m.s.d.= 1.717 Å over 331 aligned Cα-atoms, **Supplementary Figure 6d**).’

3. Please include EM densities for some representative regions of the deposited structure in the supplementary figure.

While we had presented insets depicting EM densities of the site 1 and site 2 interfaces in Figure 1D and Figure 1E, respectively, we have now added a new **Supplementary Figure 3** depicting additional and larger insets of site 1 (ALK-ALKAL2), site 2 (ALK’-ALKAL2) and site 3 (ALK-ALK’) interfaces of the 2:1 ALK-ALKAL2 cryo-EM map.

We call out this figure in following paragraph (**Lines 105 – 109**):

‘The combined use of Orientation Rebalancing in CryoSPARC and 3D refinement in RELION v5 with enabled Blush regularization, resulted in a final map at 3.2 Å resolution (**Figure 1**, **Figure 2A**, **Supplementary Table 1**) displaying an impressive improvement in isotropy, as evidenced by the 10-fold increase in the cFAR score (**Figure 2B)**, and overall interpretability (**Supplementary Figure 3**).’

4. In line 114, the cFAR score should be 0.08, as shown in Fig. 3B, rather than 0.8.

This typo is now corrected in the main text.

5. In Supplementary Table 1, the number of micrographs should be 13,618, not 13.618.

This typo is now corrected in Supplementary Table 1.

**Reviewer #4 [identifies himself as Arjen J. Jakobi]:**

This study investigates the structural mechanisms of ALK (Anaplastic Lymphoma Kinase) and LTK (Leukocyte Tyrosine Kinase) activation by their cytokines, ALKAL2 and ALKAL1, which have critical roles in development, metabolism, and cancer. Different stoichiometries of ALK/LTK-cytokine assemblies (2:1 and 2:2) have been observed via X-ray crystallography and cryo-EM; one set of original crystallography data were reported by the authors of this study. A reanalysis of the published cryo-EM data from another research team (EMPIAR-10930) by the authors revealed that 2:1 and 2:2 complexes co-occur in the published dataset and that many (in fact, a majority of) particles correspond to the 2:1 ALK-ALKAL2 complex, though these suffer from orientation bias and associated resolution anisotropy. Using recent advances in cryo-EM data processing, including a data-driven regularised refinement technique, the authors reconstructed the 2:1 ALK-ALKAL2 complex at ~3.2 Å overall resolution from the publicly deposited cryo-EM dataset. This work reconciles the structural understanding of ALK and LTK receptor dimerization and, while acknowledging both 2:1 and 2:2 complexes do exist, proposes a unifying signaling-ready 2:1 model across both systems.

The (re)analysis and the study are thorough and solid, carefully performed and presented, and the manuscript is well written and illustrated. I enjoyed reading it. The authors present a series of structurally convincing arguments why the 2:1 complex appears to be a plausible arrangement also in a physiological context. The study not only adds a new (=more complete) 2:1 structure to the collection of experimentally determined structures of the ALK-ALKA2 complex, but also provides a beautiful example why public deposition of raw electron micrographs is so important for the community to allow re-analysis of previous data while computational tools develop and improve. I am limiting my comments to technical aspects regarding the analysis of cryo-EM data and data processing workflows as I lack expertise related to cytokine biology that would allow me to evaluate the biological conclusions drawn. Below, I summarize a few comments and suggestions that the authors may consider.

We thank the reviewer for the careful assessment and endorsement of our work and the constructive input.

Minor comments:

1. A host of data and experiments suggest that unbalanced particle orientations in cryo-EM single particle samples can - more often than not - be attributed to preferential adsorption of the molecules at the air-water interface (AWI) of holey support films. Whilst the authors' analysis in the present study is perfectly solid and the evidence for a 2:1 complex in the EMPIAR-10930 data is unambiguous, the possibility remains that the observed 2:1 complex, which displays strong orientation bias, could also be a result of 2:2 complexes partly dissociated or otherwise structurally biased at the AWI. I acknowledge that this would involve a substantial conformational change to accommodate the relative orientation of both ALK monomers in the observed 2:1 ALK-ALKAL2 complex vs the 2:2 complex, which may not be obvious (and possibly unlikely) but as far as I infer from the accumulated data can also not be decisively refuted a priori.

While this comment raised by the reviewer is certainly a valid concern, we would like to argue that association/adsorption at the air-water interface (AWI) does not necessarily imply aggregation or structural bias. The ALKAL2(C66Y) construct expressed in Reshetnyak *et al.,* Nature, 2021, contains an N-terminal extended tail with an amphipathic α-helix, which is not visible in the 2:1 or 2:2 ALK-ALKAL2 complex 3D reconstructions obtained from the EMPIAR-10930 dataset due to its inherent flexibility. Due to the differences in assembly between 2:1 and 2:2 ALK-ALKAL2 complexes, it may be possible that the N-terminal tail of ALKAL2(C66Y) interacts more easily with the air-water interface in the 2:1 complex when compared to the 2:2 complex. Interaction of the sideways protruding N-terminal tail of ALKAL2 with the air-water interface would result in a 2:1 ALK-ALKAL2 side view, which happens to be the most abundant orientation of the 2:1 ALK-ALKAL2 complex in the EMPIAR-10930 dataset. Furthermore, the 2:1 ALK-ALKAL2 complex contains a second ALK-ALKAL2 interface (630 Å^2^) that is not compatible nor overlapping with the second ALK-ALKAL2 interface found in the 2:2 ALK-ALKAL2 assembly (250 Å^2^). If applicable, disassembly of the 2:2 complex at the air-water interface would likely lead to two 1:1 ALK-ALKAL2 complexes with an intact high affinity site 1 interface (755 Å^2^), common to 2:1 and 2:2 ALK-ALKAL2 complexes. Therefore, while technically not impossible, the proposed scenario where a 2:2 ALK-ALKAL2 complex disassembles at the air-water interface into a 2:1 ALK-ALKAL2 complex would seem highly unlikely in our opinion. Moreover, we would like to iterate that the conservation of the first and second receptor-ligand interaction interfaces as well as the major receptor-receptor contact interface in evolutionary related 2:1 ALK-ALKAL2 and LTK-ALKAL1 complexes strengthens the putative physiological relevance of the 2:1 ALK-ALKAL2 assembly in signaling.

We have added the latter statement in the Introduction of the manuscript (**Lines 40 – 43**):

‘In accordance with the evolutionary relatedness[8] and close structural similarity between ALK and LTK, key receptor-receptor and receptor-ligand interactions are conserved in the resulting 2:1 stoichiometric ALK-ALKAL2 and LTK-ALKAL1 assemblies[5], which are pseudo-symmetric due to the asymmetric nature of the ALKAL2/1 ligands.’

Additionally, we would like to point out that full-length wild type human ALKAL2 can form a dimer in solution via Cys66 in the predicted α-helix of its flexible N-terminal tail, yet monomeric ALKAL2 lacking this tail is as active as its dimeric counterpart (Reshetnyak *et al*. 2015, PNAS). While our study by De Munck *et al.* and the study by Reshetnyak *et al.* used monomeric or mutated (C66Y) ALKAL2, respectively, both 2:1 and 2:2 ALK-ALKAL2 assemblies revealed from our reanalysis of EMPIAR-10930 are structurally compatible with dimeric ALKAL2. In the 2:2 ALK-ALKAL2 assembly, the additional 2 x 68 N-terminal ALKAL2 residues not built in the cryo-EM structure would need to be tacked underneath or along the length of the complex parallel to the cell membrane. On the other hand, in the 2:1 ALK-ALKAL2 assembly the N-terminal tail would have ample space to flare outwards, allowing its facile linkage to another 2:1 ALK-ALKAL2 complex. We have added the following to the discussion of the manuscript (**Lines 188 – 196)**:

‘Full-length wild type human ALKAL2 can form a dimer in solution via Cys66 in the predicted α-helix of its flexible N-terminal tail, yet monomeric ALKAL2 lacking this tail is as active as its dimeric counterpart [2]. While our study by De Munck *et al.*[5] and the study by Reshetnyak *et al.*[7] used monomeric or mutated (C66Y) ALKAL2, respectively, both 2:1 and 2:2 ALK-ALKAL2 assemblies present in EMPIAR-10930 are structurally compatible with dimeric ALKAL2. In the 2:2 ALK-ALKAL2 assembly, the additional 2 x 68 N-terminal ALKAL2 residues not built in the cryo-EM structure would need to be tacked  underneath or along the length of the complex parallel to the cell membrane. On the other hand, in the 2:1 ALK-ALKAL2 assembly the N-terminal tail would have ample space to flare outwards, allowing facile linkage to another 2:1 ALK-ALKAL2 complex.’

Another slightly worrying observation is that out of almost 12,000,000 cleaned-up particles after several rounds of 2D classification only about 10% (~870,000 for 2:1 + ~520,000 for 2:2) remain after 3D (hetero)refinement (cf. Figure 2A), which might suggest that a majority of particles is either structurally compromised or otherwise limited in contributing useful signal. Are all the discarded particles associated with "junk" classes? If so, what may this tell about the sample as a whole? If not, how were the final classes selected? None of the above observations question or invalidate the authors' conclusions in any way, but I invite the authors to consider and discuss these possibilities; or discuss why they can be discarded.

The discarded particles were not at all associated with “junk” classes, since these were already removed in the first 2D classification using 18 million 4x binned particles. After particle unbinning of 11.8 million remaining particles (cleaned particle stack), *Ab Initio* model generation and ensuing 3D heterorefinement, we merely selected the 3D classes that corresponded to the highest resolution, and grouped these in two pools based on the presence or absence of twofold symmetry. Although the possibility remains that we were too stringent in 3D class selection and therefore discarded potentially useful particles, we note that we did not disregard classes that looked noticeably different from the 2:1 and 2:2 ALK-ALKAL2 assemblies present in the EMPIAR-10930 dataset (in order to not bias the workflow), and that the two obtained final maps are at a high enough resolution and overall quality to allow *de novo* model building.

2. My previous question is also spurred by the authors' earlier observation of the 2:1 ALK-ALKAL2 complex in the crystal (PDB 7NWZ) but not in solution with SEC-MALS experiments in their original report (Munck et al., 2021); instead, a 1:1 complex was the abundant species in solution, in agreement with ITC experiments that also were consistent with 1:1/2:2 binding. In contrast, as observed in with the crystal structure, the cryo-EM data appears to suggest that the 2:1 stoichiometry is the dominant complex under these conditions. Are these, on first glance conflicting, observations unique to ALK-ALKAL2 or have similar intricacies also been observed in other cytokine/cytokine receptor systems? If the authors consider the cryo-EM experiments as representative for the behavior in bulk solution, how do can the the biochemical data be congruently rationalised with the observations from crystallography/cryo-EM? This is intriguing and some discussion to place these observations in context would be helpful to readers with limited knowledge of the cytokine field like me.

Our SEC-MALS and ITC studies of the ALK-ALKAL2 interaction have revealed that the binding affinity of site 2 observed for the ALK-ALKAL2 complex cannot be supported at the concentrations used for these experiments. As we commented in our original work by De Munck *et al.* 2021, “In hindsight, the inordinately high protein concentration reached during crystallization might have compensated for the low degree of cytokine-induced ALK_TG_ dimerization in solution.” Furthermore, structural comparisons with the related LTK-ALKAL1 complex had established that site 2 in the case of the ALK-ALKAL2 complex does not display the hydrophobicity grade of the equivalent site 2 observed in the LTK-ALKAL1 complex. Additionally, receptor-receptor contacts composing site 3 are more extensive in the LTK-ALKAL1 complex, featuring more extensive interactions. Thus, sites 2 & 3 of the ALK-ALKAL2 complex are intrinsically much weaker than for LTK-ALKAL1, in accordance with the comparative ITC and SEC-MALS data shown for the two systems.

Due to the approach and high concentration used during sample preparation in the cryo-EM study by Reshetnyak *et al.*, the 2:1 complex requiring engagement of site 2 was enriched, analogously to our original crystallographic studies.

Structure-function studies of cytokine-receptor signaling mechanisms have shown that many cytokines contain both a high-affinity and low-affinity receptor binding site. The co-existence of a strong site 1 versus a weak site 2 has been thought to be part of how signaling can be regulated as a function of cytokine concentrations near the cell surface and the relative abundance of expressed receptor chains at the cell surface. This has presented the field with many challenges, and in fact many such cytokine-receptor complexes remain structurally unresolved, or have only been partially elucidated, e.g. binary complexes rather than ternary cytokine-receptor assemblies. To this end, recent methodological developments that enable the facile creation and selection of mutant variants of cytokines and receptors allowing for the maturation of such otherwise weak interaction interfaces (e.g. yeast display), have potentiated their capturing into stable complexes for structure elucidation by cryo-EM and X-ray crystallography.

3. While the improvement of RELION refinement is evident, I feel it would be good to separate out the effect of Blush regularisation from regular RELION refinement to determine if indeed it is the additional regularisation that is beneficial/necessary in this case, since from Figure 3B it appears a major factor was orientation re-balancing. Currently the authors compare NU refinement in Cryosparc to Blush regularisation in RELION for particle sets before and after orientation re-balancing. The beneficial effect of RELION/Blush is most striking for the case before orientation re-balancing. I agree with the authors that comparing Cryosparc NU refinement to RELION+Blush is the most meaningful comparison as both strategies employ fundamentally similar, though conceptually different, ways of refinement regularisation. To separate out the effect of Blush regularisation over regular RELION refinement on improving isotropy of the reconstructed map, it should ideally be compared to a RELION 3D refinement without Blush regularisation using the same orientation-rebalanced particle set. Such an analysis would strengthen the assertion that the observed effect is indeed due to improved regularisation through Blush during refinement as opposed to a more generic difference in CryoSparc NU vs. regular RELION refinement.

Indeed, 3D refinement starting from the Orientation Rebalanced particle set, but without enabling Blush regularization, was missing from our analysis presented in Figure 3. We have now performed this additional refinement, by running an exact copy of the RELION run that lead to the final map of the 2:1 ALK-ALKAL2 complex, but now with disabled Blush regularization. This 3D refinement resulted in a more isotropic 3D reconstruction (cFAR score of 0.25) than NU-refinement in CryoSPARC after Orientation Rebalancing, however, it finishes with a far lower resolution of 4.3 Å, which does not allow unambiguous *de novo* model building.

We have updated Figure 3 with this additional refinement, and have accordingly updated the final paragraph of the main text (**Lines 134 – 143**):

‘Furthermore, we also performed a 3D refinement in RELION starting from the Orientation Rebalanced particle set, but without enabling Blush regularization (Figure 3A & B). This refinement resulted in a more isotropic 3D reconstruction (cFAR score of 0.25) than NU-refinement in CryoSPARC after Orientation Rebalancing, but stalled at a lower resolution of 4.3 Å, prohibiting *de novo* model building. Thus, enabling Blush regularization during 3D refinement of the Orientation Rebalanced particle set seems to both result in increased map isotropy as well as resolution. Taken together, our analysis demonstrates that the combination of extensive Orientation Rebalancing and 3D refinement with Blush regularization may serve as a suitable strategy to overcome severe preferred particle orientations *In Silico* in datasets that contain a full but extremely unbalanced range of orientations.’

4. Page 3: "...of similar resolution and informational content": I believe it should be "information content".

This has now been corrected in the main text.

5. Page 3: "...a massively improved final map...": it is ambiguous what "massively improved" means or refers to. I suggest either dropping the adjective or making explicit what property/properties of the map has/have been improved (isotropy, interpretability, ...).

We have changed the sentence accordingly (**Lines 105 – 109**):

‘The combined use of Orientation Rebalancing in CryoSPARC and 3D refinement in RELION v5 with enabled Blush regularization, resulted in a final map at 3.2 Å resolution (**Figure 1**, **Figure 2A**, **Supplementary Table 1**) displaying an impressive improvement in isotropy, as evidenced by the 10-fold increase in the cFAR score (**Figure 2B)**, and overall interpretability (**Supplementary Figure 3**).’

6. Caption Figures 1,2; Supplementary Figures 2: "Gold-standard Fourier Shell Correlation (FSC) plots corresponding to the final maps...". Whilst broadly used, the term "gold-standard FSC" is not without controversy in the cryo-EM field. A neutral way could be simply stating: "Fourier Shell Correlation (FSC) plots computed from independently refined half maps of...".

We have removed the two instances of ‘Gold-Standard’ in the legends of Figure 1 and Supplementary Figure 2, and have changed the mentioning of ‘Gold-Standard’ in the Materials and Methods section as follows (**Lines 243 – 245**):

‘The final obtained map has a Fourier Shell Correlation (FSC, computed from independently refined half-maps) resolution of 3.2 Å at the 0.143 threshold (**Figure 1, Supplementary Table 1**)’
